# Behavior of Pigs Reared in Enriched Environment: Alternatives to Extend Pigs Attention

**DOI:** 10.1371/journal.pone.0168427

**Published:** 2017-01-06

**Authors:** Simone Pereira Machado, Fabiana Ribeiro Caldara, Luciana Foppa, Rafael de Moura, Liliane Maria Piano Gonçalves, Rodrigo Garófallo Garcia, Irenilza de Alencar Nääs, Viviane Maria Oliveira dos Santos Nieto, Geyssane Farias de Oliveira

**Affiliations:** 1 College of Agrarian Sciences, Federal University of Grande Dourados, Dourados, Mato Grosso do Sul, Brazil; 2 Center of Agrarian Science, State University of Londrina, Londrina, Paraná, Brazil; Universita degli Studi di Pisa, ITALY

## Abstract

Three trials were carried out in a completely randomized design aiming to assess the behavior of pigs in growth phase in enriched environments. Trial 1 evaluated the effects of frequency of availability of environmental enrichment. The animals were assigned to four treatments: 1) control with no enrichment object; 2) objects provided for six consecutive days uninterruptedly; 3) objects provided on alternate days, and 4) objects provided for six consecutive days taken away by the end of the afternoon and replaced at dawn. Trial 2 assessed the effects of scent on animals’ acceptance and maintenance of interest in objects. Animals were assigned to four treatments: 1) unscented object; 2) object with banana scent; 3) object with rum scent; 4) object with scents alternated every other day. Trial 3 aimed to assess the influence of environmental enrichment based on providing rewards at different difficulty levels. Animals were assigned to three treatments: 1) object with no reward; 2) object with a reward at an easy level; 3) object with a reward at a difficult level. Each trial had six days of behavioral observations every ten minutes for eight hours each day using images from video cameras. Enrichment objects stimulated the animals’ natural behavior of nuzzling and exploring the environment. The way the objects were available did not impact the success of their use. Offering enrichment on alternate days or removing the objects by the end of the day was not an effective strategy to extend the animals’ interest. The olfactory stimulus in environmental enrichment objects had no positive effect on extending the animals’ interest on them, nor did alternating the aromas. The tactile stimulus was a key factor for object attractiveness. Providing environmental enrichment objects with rewards stimulated the exploratory behavior of pigs. The level of difficulty to obtain the reward may discourage the animals.

## Introduction

Brazilian pig farming has a predominance of intensive production systems in confinement featuring facilities with cement floors and high animal density. However, this type of system has been questioned due to the volume and difficulties in properly managing waste, which entails serious environmental pollution issues, and due to reduced animal well-being [1]. Abnormal behaviors are commonly observed in animals reared in confinement since the inability of exercising the species natural behaviors significantly impacts their well-being.

One of the most relevant subjects in welfare guidelines is the issues related to intensive confinement. In this context, Machado Filho and Hötzel [2] suggest two approaches to improving animal well-being: alternative production systems and the environmental enrichment.

Environmental enrichment is a principle of animal management that seeks to enhance the quality of life of animals in captivity by identifying and providing environmental stimuli required for them to reach psychological and physiological well-being. This strategy stimulates the behaviors typical of the species, thus lowering stress and making the captive environment more complex and diverse by meeting their ethological needs [1]. According to Campos [3], environmental enrichment is a way of providing better living conditions to the animals. Pigs reared in enriched environments typically show behavioral evidence of better well-being compared to those in confinement [1] and, when the strategies are well planned and applied, they may significantly reduce the frequency of abnormal behaviors [4].

One way of enriching the environment is by including objects designed to attract interest and allow the animals to express species-specific behaviors. Current literature suggests the environmental enrichment objects must be attractive, chewable, consumable, deformable, destructible, and have a stimulating scent or flavor, and yet create novelty [5]. Those objects must motivate the species and prove effective in their goal throughout the production process since studies indicate that the animals quickly lose interest in them [6, 7]. Studies have shown the reduction in pig interaction with the objects within five to six days [8, 5].

Thus, the current challenge for the scientific community is to develop environmental enrichment techniques that meet their goals of improving animal well-being, and that might maintain continued interest in the objects while being easily implemented and economically viable.

The current study was developed aiming to assess the behavior of pigs in the growth phase in enriched environments, as well as to evaluate alternatives that aim to extend animal interest in the objects used as enrichment.

## Material and Methods

Three trials were carried out between September and October 2015 on a commercial pig farm in the city of Dourados, MS, Brazil. The city’s coordinates are 22°13’18.54” S and 54°48’23.09” W and its main altitude is 430 m. The climate in the region is humid mesothermal (Cwa) according to the Köppen classification featuring rainy summers and dry winters with an average annual rainfall of 1,500 mm and average annual temperature of 22°C. The study was done during the day in a way not to interfere with the general farm management.

The research complied with ethical standards and was approved by the Ethics Committee on Animal Use (permit 06/2015) of the Federal University of Grande Dourados.

The study used 506 commercial hybrid grower pigs (161 females and 345 males) all of the same genetic lineage. The trials were carried out before the immunocastration of the male grower pigs. The animals were housed in a masonry barn (100 m long x 8.0 m wide) with collective 60 m^2^ pens featuring a running water pool at the end of the pen, automated feeding troughs, and nipple drinker. Each pen housed around 46 animals whose mean initial weight was 25 ± 2 kg. The animals remained 21 days adapting to the facilities and establishing social hierarchy and the experimental evaluations began when they were 86 days old. The pigs were fed and had access to water *at libitum*.

### Trial 1

The trial was carried out aiming to assess the behavior of pigs in an environment enriched with objects and the effects of the frequency of availability of those objects on the maintenance of interest by the animals. A total of 184 animals (92 females and 92 non-castrated males) were assigned to the four treatments in a completely randomized design with 46 animals per treatment:

T1- control treatment with no enrichment object;
T2—objects provided uninterruptedly for six consecutive days;
T3- objects provided on alternate days over six days;
T4- objects provided in the morning and taken away by the end of the afternoon for six consecutive days.

The objects tested in T4 were removed immediately after the video recording from the pen every day at 3:15 PM and added again in the next day at 6:30 AM. The video recording only started 40 minutes after the tested objects were added to the pen to avoid the change in the behavior of the pigs due to the presence of the researcher inside the pen.

**Fig 1:**
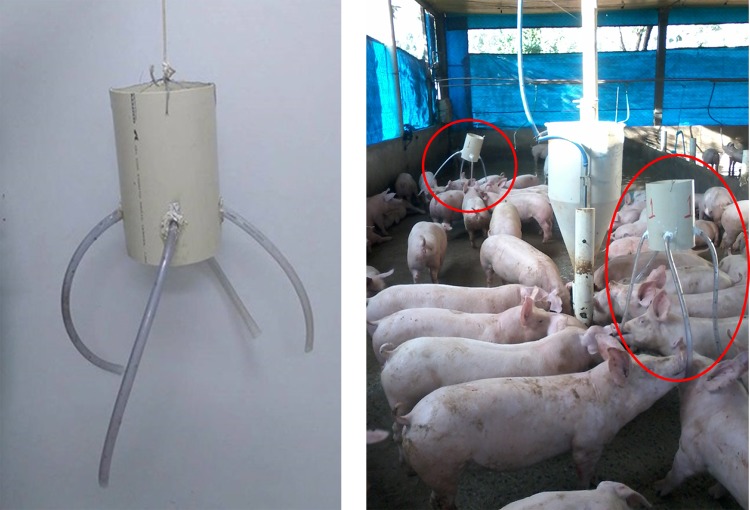
Environmental enrichment object. Environmental enrichment objects hung at the animals’ eye height.

The environmental enrichment objects were made with PVC pipes 25 cm long and 20 cm in diameter attached to four 65 cm-long pieces of non-toxic clear plastic tubing, which allows the pigs to exercise the exploratory activity of chewing. Each length of pipe was filled with sisal ropes to absorb the impact of the animals’ bites, thus keeping them from breaking. Two objects were hung in each pen at the pigs’ eye height to facilitate visual contact, except in the control treatment (Fig 1).

### Trial 2

The trial was carried out aiming to assess the animals’ acceptance of scented enrichment objects, as well as the effects of alternating scents on the extension of the pigs’ interest in the objects. A total of 184 animals (non-castrated males) were assigned to four treatments in a completely randomized design with 46 animals per treatment:

T1- unscented enrichment object (control);
T2- banana-scented enrichment object;
T3- rum-scented enrichment object;
T4- banana- and rum-scented enrichment object alternated every other day.

**Fig 2:**
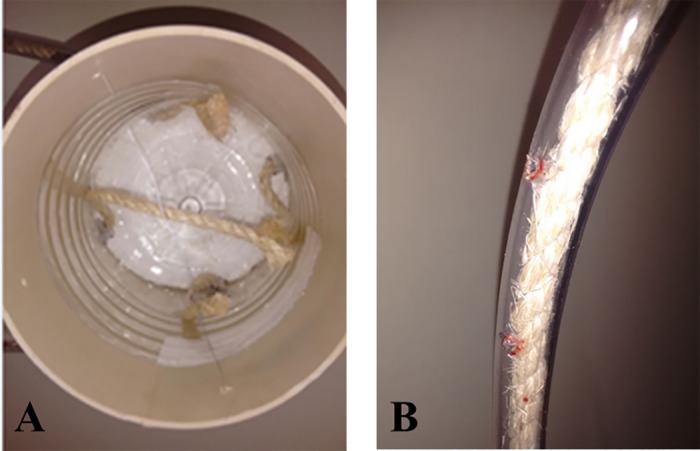
Interior of the enrichment object with a compartment to store the essence (A) and holes in the plastic tube (B).

Two objects were hung per pen in the same way as in trial I. Holes were drilled in the plastic pipe attached to the PVC structure. The aromatic essences (Arcolor®; Composition: artificial aromatic basis diluted in water and neutral ethylic alcohol) were stored inside the PVC pipe and were absorbed by the sisal ropes and exhaled through the holes in the plastic tube (Fig 2). The essences inside the PVC structure were refilled daily to keep the scent durable. The essences were diluted in water at 0.1:1 ratio (100 mL essence for 1.0 L water). The scents presented were based on the human perception of odor believing their stimulus value to a pig, since these animals have a very good sense of smell [9]. The authors indicate pigs present an olfactory acuity approximately 2000 times more sensitive than human; therefore, the dilution proposed in the present study aimed to be easily detected by the pigs. The objects were hung at the pigs’ eye height to enable visual contact. The essence was alternate (banana- and rum-scented) every day during T4 in the attempt to keep each odor as a novelty for the pigs.

### Trial 3

**Fig 3:**
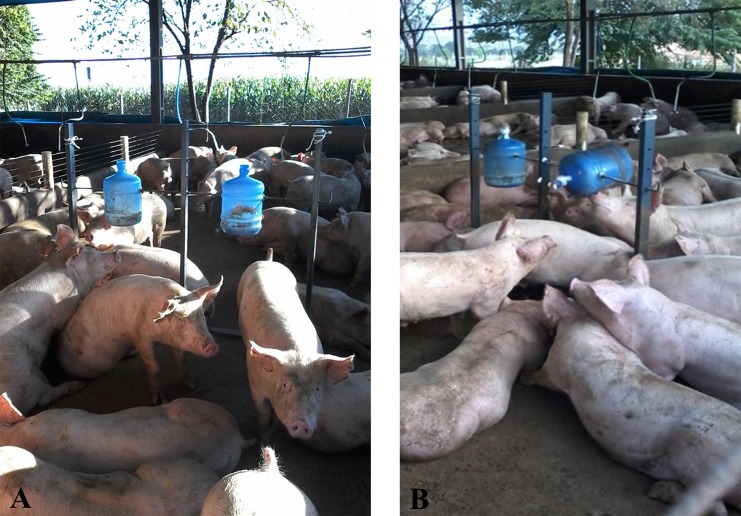
Environmental enrichment object made with jugs attached to the steel structure (A); Animals are interacting with the environmental enrichment object (B).

The trial aimed to assess the pigs’ behavior in the presence of environmental enrichment objects based on providing rewards (tasty snacks) at different difficulty levels. The animals (n = 138–69 females and 69 non-castrated males) were assigned to three treatments in a completely randomized design with 46 animals per treatment in the same pen. The treatments were:

1. Providing an enrichment object with no reward;
2. Providing an enrichment object with reward at an effortless level;
3. Providing an enrichment object with reward at a challenging level;

The enrichment objects were made using empty 10 L water jugs, and steel stands 1.15 m tall x 1.45 m wide. A steel rod was placed across the jugs and attached to the stand. Two jugs were placed in each rod, forming a spinning structure (Fig 3).

The structures were fixed to the floor of the pens in a way that the jugs were at the height of the animals’ heads not to be touched. The interaction of the pigs with the toy should make the jugs spin 360° to release the reward inside. Different holes were drilled in the jugs to customize the levels of difficulty to turn them so the reward could be obtained. Therefore, the animals in the treatment with difficult level had to try harder to be able to turn the jug and get the reward.

The reward consisted of popcorn, which was shown to be very attractive to the pigs in a previous pilot experiment. The snack was also a regular part of the animals’ diet (corn), so they would pose no risk. The popcorn was made and replaced every morning before the behavioral observations.

### Behavior Analyses

All observations were performed for a total of 8 h daily, between 7:10 AM and 3:10 PM, during six consecutive days during the experimental period. The pig behavior was analyzed using video images recorded by video cameras installed at the top of the pens and directly connected to a device with an image capture card and LCD monitor. One video camera was installed for each treatment, with independent adjustments, targeting the objects and focusing the whole pen. The video images were stored in the monitoring apparatus’s internal memory for later evaluation.

The video images were watched using the video software CyberLink and the recording was paused every 10-min completing 48 events per day (480 minutes of video recording per day).

**Fig 4:**
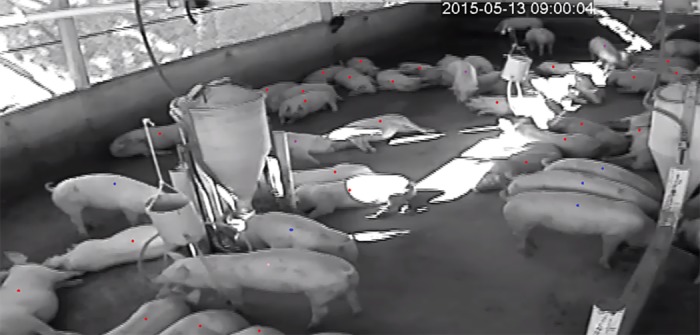
Marking the animals with different colors to identify the behaviors listed in the ethogram.

The video image instantaneous scan samples were taken using the print screen function on the computer. The instantaneous scan samples were opened in the software Paint (Microsoft Windows^®^ operational system) and then marked with colors (Fig 4) that represented each animal behavior listed in the ethogram (Table 1). All animals in each pen were evaluated individually, and the number of the animals performing each behavior was registered and transposed to a spreadsheet.

**Table 1:** Behavioral ethogram used to assess pigs in growth housed in environments with different environmental enrichment strategies.

Behavior	Identification		Description
**Stance**			
Sleeping or lying	Red		Animal lying with the body touching the floor or stretched on it with eyes closed or open
Moving around or sitting	Purple		The slow movement is walking in the pen. Sitting.
**Interaction with the environment**			
Eating or drinking	Blue		Pig with the head by the drinking trough or feeding trough
Nuzzling or exploring the environment	Black		Exploratory function, investigating the environment, nuzzling, sniffing or biting some element of the pen
Interacting with the object	Pink		Sniffing, biting or nuzzling the enrichment object
**Social interaction**			
Sexual behavior		Brown	Mounting or letting a partner mount
Agonistic behavior		Orange	Confrontation, headbutts, fights, and chasing a partner in the pen
Interaction with a partner		White	Nuzzling some part of another pig, playing

Since the trials were carried out on a commercial farm, the experiments were done with the minimum interference in the general management generating a limitation to perform the tests during the day. The trials were carried out during the Southern hemisphere winter with short photoperiod when the pigs have their peak of activities close to the time chosen to the monitored.

The behavioral assessment used an ethogram adapted from the methodology proposed in the literature [3,10] (Table 1).

### Statistical Analysis

Activity behavior parameters measured the number of pigs per pen performing a particular activity in each event (48 events per day) registered every 10 minutes. A histogram of the frequency of behavioral activities was built. Activity behaviors were recorded as the percentage of pigs performing a particular behavior at each scan sampling occasion and were analyzed as a percent of the time [11]. For each day of observation, there were 48 events, and for each event, it was considered that the pig exerted that particular behavior until the next event (10 minutes) [11].

The mean values concerning each behavior present in the ethogram were determined in minutes and as percentages of the total time. The time in minutes the animals spent in each assessed behavior was calculated based on the average of 46 animals per treatment. The frequency was considered as a percent of the total evaluated time. The values presented were the average number of a given behavior in six days.

Data normality was verified using Lilliefors test, analysis of variance was applied, and the means were compared by Tukey’s test using the software Assistat [12].

## Results and Discussion

### Trial 1

**Fig 5:**
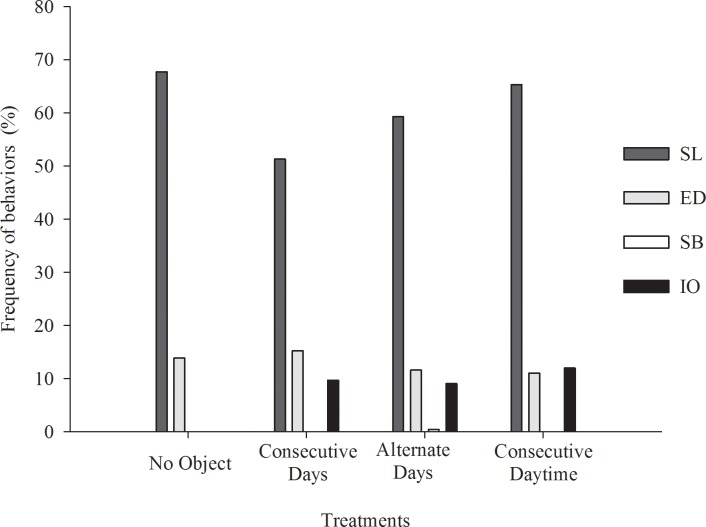
Impact of different environmental enrichment strategies on behaviors of pigs. SL = sleeping or lying; SB = sexual behavior; ED = eating or drinking; IO = interacting with the object; (Fig 5 data is in S4 File).

The different methods of providing the environmental enrichment objects did not impact (p>0.05) the time the animals spent in agonistic behaviors, nuzzling and exploring the environment, interacting with the enrichment object or with other pigs, or moving around or sitting (Table 2) (Fig 5).

**Table 2:** Time (min)* and behavioral frequency (%) of pigs in growth in an environment enriched with objects provided using different methods.

	Treatments								
	No object		Consecutive days		Alternate days		Consecutive daytime		Significance
Behavior	min	%	min	%	min	%	min	%	
SL	325.06	67.72a	279.89	58.31c	284.50	59.27bc	313.49	65.31ab	**
ED	66.62	13.88a	73.10	15.23a	55.63	11.59b	52.80	11.00b	**
SB	0.38	0.08b	0.05	0.01b	2.02	0.42a	0.38	0.08b	**
AB	2.93	0.61	1.73	0.36	4.75	0.99	1.49	0.31	NS
NE	69.70	14.52	55.49	11.56	74.06	15.43	47.86	9.97	NS
IO	0.00	0.00	46.51	9.69	43.44	9.05	57.50	11.98	NS
IP	0.14	0.03	1.87	0.39	2.16	0.45	0.14	0.03	NS
MS	15.17	3.16	21.36	4.45	13.44	2.80	6.34	1.32	NS
Total	480	100	480	100	480	100	480	100	-

SL = sleeping or lying; SB = sexual behavior; AB = agonistic behavior; ED = eating or drinking; NE = nuzzling or exploring the environment; IO = interacting with the object; IP = interacting with another pig; MS = moving around or sitting.

* Time in minutes spent in each behavior for 8 h daily (480 min per day) (mean of the six days of observation).

Means followed by small letters on the rows differ according to Tukey’s test.

** Significant at 5% probability level; NS = non-significant. (Table 1 data is in the S1 File)

The animals spent on average over 60% of the time sleeping or lying. The result is similar to those from a previous study [13] that states that pigs spent on average 61% of the time sleeping in an environment with no enrichment. Compared to all other farm animals, pigs spend the most time idle and sleeping [14]. However, the pigs maintained in environments with no environmental enrichment (control) spent more time resting (p<0.05) compared to those that had the objects constantly in the pen (T2) or yet with objects on alternate days (T3).

The animals in the treatments with no objects and with objects constantly in the pen for six consecutive days spent more time eating and drinking than the others. In a study where the performance of pigs reared in the enriched and not enriched environment, it was found that high daily intake in the group in the barren environment had no positive impact on the finishing animals’ performance [15]. The authors credited this result to the waste of feed by the pigs that used the food as a playing source.

The pigs in the treatment in which the object was provided on alternate days had a higher frequency of sexual behavior (p<0.05). Sexual behaviors, such as mount, are part of the natural behavior repertoire of pigs [16]. However, some factors may change the natural frequency of those behaviors, and that may become a problem for the animals’ well-being. There is no apparent explanation for only the animals in that group showing a greater expression of sexual behavior. Nonetheless, the expression did not reach high levels that could compromise the animal welfare. This behavior was found in the range of 1.2 to 2.0% in a previous study that evaluated the effectiveness of an immunocastration vaccine to suppress aggressive and sexual behaviors of entire male pigs [11].

Agonistic behaviors are also part of the standard behavior of pigs. It is known that pigs are animals that fight over the control of resources or space and perform shows of force. The frequencies observed for agonistic behavior were relatively low at 0.49% on average of the total period assessed. Neither the enrichment objects nor the strategies of providing them were able to reduce this behavior compared to the control treatment. Other investigations also found no effect of environmental enrichment on the reduction of aggressive behaviors [17, 18].

Environmental enrichment did not impact (p>0.05) the expression of investigative behavior in the animals. Pigs have the natural habit of nuzzling and exploring the environment. In natural conditions, these animals spend much of their time searching for food and exploring the environment and this type of behavior is considered crucial for the species [19, 20]. In the present study, the animals spent around 10% to 15% of their time exploring the environment. That shows that the confinement environment, even when enriched, limits the expression of the natural animal behavior and that the objects in the pen may have diverted part of the animals investigative behavior.

**Fig 6:**
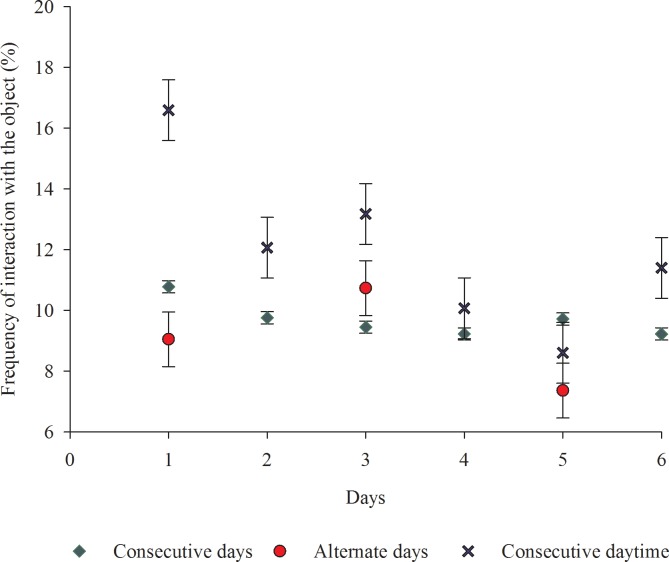
Frequency (%) of animal interaction with the enrichment objects over the six experimental days. *Consecutive daytime = objects provided for six consecutive days taken away by the end of the afternoon and replaced at dawn. (Fig 6 data is in S5 File).

The time the animals spent interacting with the enrichment objects showed that they were attractive and well accepted by the animals, which may be related to the characteristics of the materials employed. The flexibility and destructibility of the object used as environmental enrichment positively contribute to the success of the technique [21]. However, the different frequencies of providing the objects did not impact (p>0.05) the time the animals spent interacting with the object. An important property of an enrichment object is to generate the feeling of novelty involved in exploration [22]. However, little is known about the time pigs take to forget a particular object. It was expected that providing the objects on alternate days or taking them away at dusk and replacing them at dawn might stimulate greater interest by the animals in them and become an effective strategy to extend the benefits of enrichment. However, that was not observed and the time the animals spent interacting with the objects was similar among the treatments, as well as the frequency over the experimental period (Fig 6).

### Trial 2

**Fig 7:**
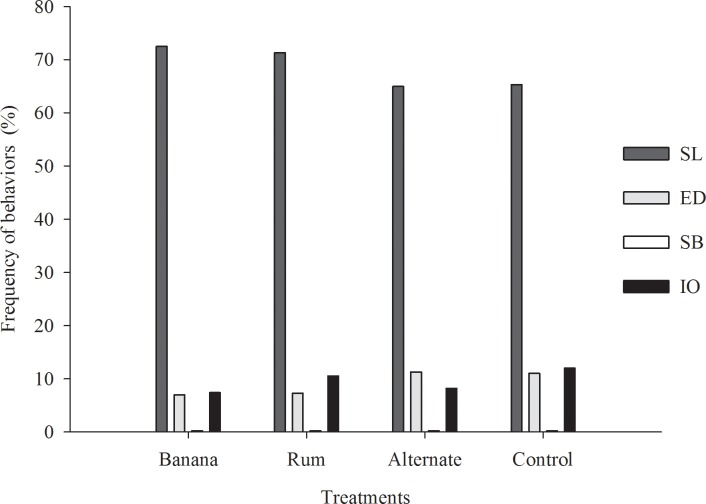
Impact of flavored environmental enrichment objects on behaviors of pigs. SL = sleeping or lying; SB = sexual behavior; ED = eating or drinking; IO = interacting with the object; (Fig 7 data is in S6 File).

The different treatments did not impact (p>0.05) the time the animals spent with the behavioral activities: sleeping or lying, sexual and agonistic behavior, nuzzling or exploring the environment, moving around or sitting, and interacting with the other pigs (Table 3) (Fig 7).

**Table 3:** Time (min)* and behavioral frequency (%) of pigs in growth in an environment enriched with objects provided using different methods.

	Treatments								
	Banana		Rum		Alternate		Control		Significance
Behavior	min	%	min	%	min	%	min	%	
SL	348	72.5	342.29	71.31	311.90	64.98	313.49	65.31	NS
SB	0.624	0.13	0.72	0.15	0.43	0.09	0.38	0.08	NS
AB	0.768	0.16	0.82	0.17	0.67	0.14	1.49	0.31	NS
ED	33.456	6.97b	34.90	7.27b	54.05	11.26a	52.80	11.00a	**
NE	57.264	11.93	47.23	9.84	67.87	14.14	47.86	9.97	NS
IO	35.376	7.37b	50.45	10.51ab	40.70	8.48ab	57.50	11.98a	**
IP	0.192	0.04	0.19	0.04	0.24	0.05	0.14	0.03	NS
MS	4.32	0.9	3.41	0.71	4.13	0.86	6.34	1.32	NS
Total	480	100	480	100	480	100	480	100	-

SL = sleeping or lying; SB = sexual behavior; AB = agonistic behavior; ED = eating or drinking; NE = nuzzling or exploring the environment; IO = interacting with the object; IP = interacting with another pig; MS = moving around or sitting.

* Time in minutes spent in each behavior for 8 h daily (480 min per day) (mean of the six days of observation).

Means followed by small letters on the rows differ according to Tukey’s test.

** Significant at 5% probability; NS = non-significant. (Table 3 data is in S2 File).

The incidence of agonistic and sexual behaviors was low at 0.19 and 0.11% of the total time on average, respectively. Agonistic behaviors are part of the normal behavioral repertoire of pigs. However, some factors such as sex and environment may increase their incidence. Since the trial was carried out with grower pigs, the frequency of either behavior can be considered small compared to another study [23]. However, the agonistic behaviors observed did not represent destructive behaviors among the animals. Destructive behaviors can be described as those in which the pigs suffer some injury or trauma, such as biting the tail, ear, flank, and vulva [24].

Entire males are more likely to have aggressive, and sexual behavior than castrated males [25]. In an earlier study [26] it was observed that, before the second dose of the immunocastration vaccine, sensitized pigs had testosterone levels comparable to that of boars. Males that show the mounting behavior more often have lower growth rates [27]. This negative relation may reflect a harmful effect on pig well-being. Thus, the use of enrichment objects may have contributed to keeping the low incidence of both behaviors, regardless of the use of scents. Synthetic maternal pheromone and non-maternal pheromones decreased aggressive behavior and improved weight gain and the feed efficiency in groups of pigs at weaning [28, 29]. However, non-pheromonal compounds or odor-masking agents were not as efficient at reducing aggressive behavior as pheromones and did not affect the performance [30]. The use of scents was successful in reducing aggressiveness when mixing pig batches or including new individuals in the group [31].

Animals in the treatments with objects containing banana or rum scents alone spent a lower percentage of the time eating and drinking compared to the control treatments or with other scents (p<0.05). This behavior may be related to the time those animals spent sleeping (approximately 72% of the time), which was 13% above the mean of the other treatments.

The presence of scents in the enrichment objects impacted (p<0.05) the time the animals spent interacting with them. The animals in the control treatment interacted with the objects more often, with a significant difference, compared to the treatment with banana scent. Such result shows that banana smell can be considered unattractive or repellent to the animal and, thus, inhibited the access to the objects. In a research where weaned, piglets subjected to transportation stress were exposed to banana scent, and maternal pheromone, it was observed that the animals spent more time resting compared to those in the control treatment [32]. However, exposing the piglets to scents may have caused fear since those animals showed lower latency time for the flight behavior. Rum scent was better accepted, and it is likely that the lower average observed for the treatment with other scents was due to the days in which banana essence was used. Pigs are omnivorous, and scavengers and odors related to feed are usually attractive. However, the scents used did not have the attractive effect expected.

The enrichment objects were attractive and stimulated the animals’ interest in them, which was evidenced by an average of 9.6% of the total time spent interacting with the objects. This percentage becomes relevant when it is compared with the mean time the animals spent with other necessary activities such as eating or drinking (9.1% of the time) and nuzzling or exploring the environment (11.5% of the time). An environmental enrichment to be considered useful, it must be able to reduce abnormal behaviors and increase the behavior patterns considered normal for the species such as exploring, social interaction, and playing [5].

**Fig 8:**
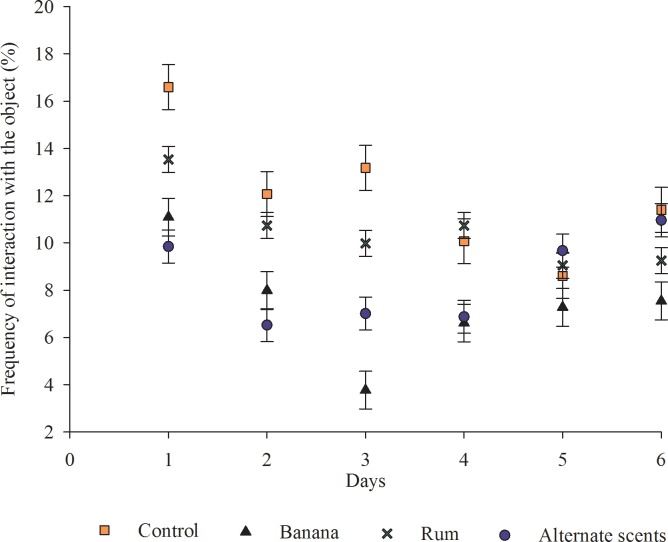
Frequency (%) of animal interaction with the enrichment objects over the six experimental days. (Fig 8 data is in S7 File).

From these results, it is inferred that the scents did not play a stimulus role, neither they impact the animals’ interest over the days (Fig 8), while the physical characteristics of the object were responsible for its attractiveness. This result matches with that reported in a previous study [8] when assessing enriched environments for pigs, the authors concluded that objects scented garlic drew less interest from the animals compared to unscented objects.

Some studies have been conducted using a wide variety of flavors to identify those most preferred by pigs. Most studies reported a preference for a sweet taste like fruity flavors [33, 34]. In previous research [5, 35], scented objects draw high initial interest that, however, in not kept over time, while unscented objects were more efficient in keeping the pig's attention. On the other hand, in another study [36] when assessing natural and synthetic scents, the author reported that weaned piglets showed a keen interest in the objects and that strawberry scent was the preferred odor. However, it is recommended a refill of the essence every 14 days to maintain a proper attractiveness.

Another study related to environmental enrichment for pigs using straw or objects like toys found that it positively impacted neural development and behavioral traits [37].

Likewise, environmental enrichment with scented elements was found to promote behavioral changes [1]. The rearing environment enriched with objects is likely to instigate pig curiosity [31]. Likewise, it is recommended the straw or any other substrate being regularly replaced as a way of increasing the animals’ interest in the enrichment factor. Cleaning the enrichment objects may also work as a strategy to stimulate the animals’ interest [38].

However, the results of the present research suggest that the attractive effect of the enriching object was caused by the object’s physical characteristics and not by the scents assessed. The hypothesis that exploring the pigs’ olfaction as a form of enrichment and that alternating scents might extend their interest could not be confirmed in this study.

### Trial 3

**Fig 9:**
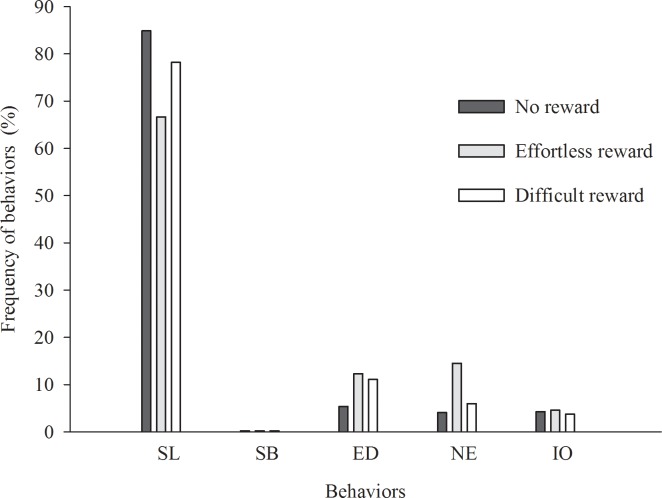
Impact on pigs’ behavior in the presence of environmental enrichment objects based on providing rewards. SL = sleeping or lying; SB = sexual behavior; ED = eating or drinking; IO = interacting with the object; NE = nuzzling or exploring the environment; (Fig 9 data is in S8 File).

The environmental enrichment objects did not impact (p>0.05) the frequency of agonistic behaviors, interaction with the object, interaction with other pigs, or moving around or sitting (Table 4) (Fig 9).

**Table 4:** Time (min)* and behavioral frequency (%) of pigs in growth in an environment enriched with objects with no reward or with different reward levels (easy or difficult) during the interaction.

	Treatments						
	No reward		Effortless reward		Difficult reward		Significance
Behavior	min	%	min	%	min	%	
SL	407.28	84.85a	319.87	66.64b	375.36	78.2ab	**
SB	0.82	0.17a	0.14	0.03b	0.29	0.06ab	**
AB	1.39	0.29	0.86	0.18	0.34	0.07	NS
ED	25.78	5.37b	59.09	12.31a	53.28	11.1a	**
NE	19.49	4.06b	69.70	14.52a	28.61	5.96ab	**
IO	20.54	4.28	21.89	4.56	17.81	3.71	NS
IP	0.10	0.02	0.10	0.02	0.10	0.02	NS
MS	4.61	0.96	8.35	1.74	4.22	0.88	NS
Total	480	100	480	100	480	100	-

SL = sleeping or lying; SB = sexual behavior; AB = agonistic behavior; ED = eating or drinking; NE = nuzzling or exploring the environment; IO = interacting with the object; IP = interacting with another pig; MS = moving around or sitting.

* Time in minutes spent in each behavior for 8 h daily (480 min per day) (mean of the six days of observation).

Means followed by small letters on the rows differ according to Tukey’s test.

** Significant at 5% probability level; NS = non-significant. (Table 4 data is in S3 File).

Regardless of the treatment, the animals spent over 65% of the time sleeping or lying. However, the animals in the treatment which offered no reward for interacting with the enrichment object spent more time idle compared to those in the treatment with effortless reward. The group dealing with difficult level (pigs had to try harder to obtain the compensation) showed intermediate frequency of interaction with the object, not differing from the others. The rates of eating or drinking behavior varied, with the animals in the treatment with no reward spending less time eating and drinking than the others, whereas they spent more time sleeping.

The presence of the reward, as well as the level of difficulty, did not impact the time the animals spent interacting with the object (p>0.05). However, the animals in the treatment with effortless reward level spent more time exploring the environment (p>0.05) compared to those that had an object with no reward (14.52% *vs*. 4.06%) which shows that providing objects with an effortless reward may have encouraged the pigs’ exploratory nature.

The exploratory behavior keeps the pigs abreast of the availability of resources, which is essential for their survival when they are dependent on exhaustible and seasonal sources of food. Although the species has been domesticated and fed by humans for several generations, the rooting appears to be a high-priority behavior. Pigs explore their surroundings by rooting, sniffing, biting, and chewing various food items as well as indigestible articles and become familiar with their environment and the different resources within it [19, 39].

The successful use of environmental enrichment is not based only on the activities targeted at the object, but also on how it may impact the pig’s behavioral repertoire. The findings suggest that environmental enrichment objects with rewards enhance the pigs’ curiosity and makes them more active during the day, thus contributing to reducing the stress generated by confinement. Pigs are opportunistic feeders and are attracted by readily available food sources, and the easy obtention of reward (popcorn) might have stimulated the exploratory behavior. However, in the current study, the increase in the difficulty in getting the compensation might have discouraged the pig's exploratory behavior (5.97%) since the animals were fed *at libitum*. Several studies show that restrictive feeding increases the occurrence of rooting behavior, *i*.*e*., appetitive foraging [40, 41] which implies that a greater motivation for exploratory behavior is to be expected in growing pigs when they are reared in feed restriction.

Although no difference was found between the frequency of interaction with the objects in the different treatments, the time the animals spent in this activity is significant at 4.18% of the total time. That makes it the fourth most exerted activity by the pigs over the assessment periods, however, it is less time than those observed in trials 1 and 2, whose objects had distinct characteristics and could be chewed by the animals.

Some authors [42–44] reported that environmental enrichment measures decrease undesirable behaviors such as aggressiveness and increase specific natural behaviors of pigs, such as investigative conduct. Environmental enrichment increases brain neurotrophin levels, which are associated with better spatial learning, memory, and manifestation of the exploratory behavior [45].

Animals in the treatment with effortless reward dedicated less time to sexual behaviors compared to those that had objects with no reward (p<0.05). The ones in the group with difficult reward showed intermediate frequency of this type of behavior. Nonetheless, irrespective of the treatment, the incidence of this behavior was low, likely because they were all female.

The relationship between the number pigs and objects used in environmental enrichment might be a limiting factor in the success of the procedure since the low ratio can lead to competition and increase of aggressivity and frustration in the reared group. A study regarding this issue [46] indicates that there was not limitation in the access to the objects by the pigs up to the ratio 1: 32 (object: pig). In the present study, there was a ratio of 1:24. The absence of agonistic behaviors in all trials of the current study indicates that the number of objects in the pen was sufficient for all pigs.

In the present study, the objects in trials 1 and 2 were placed at the height of the animals eyes, as the literature suggests that when objects offered to pigs became dirty, the pigs lost interest in them [47]. In all trials, it was noted a trend of the pigs to remain resting during the day. Confined pigs tend to sleep mostly during the day when there is no motivation for exploring [14]. Comparing the pig behavior in indoor and free-range systems [48] found that pigs in an outdoor system are more active and perform more natural behaviors (foraging and rooting) than pigs in an indoor system. In the present study using several sources of environmental enrichment, the pig behaved more similarly to those reared indoors than that in the outdoor systems. In trials 1 and 2 the pigs interacted with the objects practically the same time as feeding, indicating high importance of the enrichment presence in the pen.

Environmental enrichment devices and different strategies of exploring opportunities should be further investigated in pig production, as it appears to amend welfare conditions during rearing.

## Conclusion

The way the environmental enrichment objects were made available did not impact the success of their use. Offering enrichment on alternate days or removing the objects by the end of the day was not an effective strategy to extend the animals’ interest.

The olfactory stimulus in environmental enrichment objects had no positive effect on extending the animals’ interest on them, nor did alternating the aromas. The tactile stimulus was the key factor for object attractiveness.

Providing environmental enrichment objects with rewards stimulated the exploratory behavior of pigs. The level of difficulty to obtain the reward may discourage the animals.

## Supporting Information

S1 File Table 2 data is in S1 File. (XLSX)

S2 File Table 3 data is in S2 File. (XLSX)

S3 File Table 4 data is in S3 File. (XLSX)

S4 File Fig 5 data is in S4 File. (XLSX)

S5 File Fig 6 data is in S5 File. (XLSX)

S6 File Fig 7 data is in S6 File. (XLSX)

S7 File Fig 8 data is in S7 File. (XLSX)

S8 File Fig 9 data is in S8 File. (XLSX)
